# A Case of Multiple Myeloma Presenting with Gastrointestinal Bleeding and Evans Syndrome

**DOI:** 10.7759/cureus.5969

**Published:** 2019-10-22

**Authors:** Muhammad S Khan, Rahil Kasmani, Ghazal Khan, Khalid Changal, Hemindermeet Singh

**Affiliations:** 1 Internal Medicine, Mercy St. Vincent Medical Center, Toledo, USA; 2 Nephrology, Mercy St. Vincent Medical Centre, Toledo, USA; 3 Internal Medicine, University of Missouri, Kansas City, USA; 4 Cardiology, University of Toledo Medical Centre, Toledo, USA; 5 Cardiology, Mercy St. Vincent Medical Centre, Toledo, USA

**Keywords:** multiple myeloma, evans syndrome, immune thrombocytopenia, chemotherapy

## Abstract

Autoimmune events are rare in multiple myeloma (MM). Herein, we report a rare case of a patient presenting with recurrent gastrointestinal (GI) bleeding of unknown origin, also having pancytopenia eventually diagnosed as MM with Evans syndrome. This is an uncommon disorder presenting as autoimmune hemolytic anemia (AIHA) with immune thrombocytopenia purpura (ITP). A 56-year-old African American male presenting with recurrent GI bleeds and pancytopenia of unknown origin developed acute colonic diverticulitis on recurrent admissions, and sigmoid colectomy with primary anastomosis was performed. Flow cytometry with serum protein electrophoresis eventually revealed IgG MM with elevated Kappa/Lambda ratio. Bone marrow biopsy revealed 80% to 90% Kappa clonal plasma cells confirming MM. Direct antiglobulin test (DAT) was positive with pancytopenia. The patient initially showed a good response to chemotherapy with thrombocytopenia improving with intravenous (I/V) dexamethasone. DAT done after completion of initial chemotherapy was negative. However, his disease relapsed after three months with pancytopenia and DAT becoming positive again. The patient was restarted on chemotherapy for debulking, which resulted in a negative DAT again after two months, but pancytopenia did not improve. The patient eventually passed away due to subarachnoid hemorrhage. We highlight only this fourth reported case because of its unique presentation. In elderly patients with unknown cause of GI bleeding with pancytopenia, blood dyscrasias, especially MM, should be considered. Autoimmune workup if positive might warrant the use of steroids for pancytopenia, which can improve thrombocytopenia in MM with Evans syndrome but not anemia.

## Introduction

Anemia of variable severity is present almost universally in multiple myeloma (MM) and might be due to various causes. Several factors may contribute to the diagnosis of anemia in MM; however, the majority of cases present as anemia of chronic disease due to blunted erythropoietin production and effects of chemotherapy [1-2]. Several cases of MM along with autoimmune hemolytic anemia (AIHA) have been reported [3-5]. A prospective study investigated 66 patients with MM, out of which seven were found to have AIHA [6]. To our knowledge, only three cases of MM with Evans syndrome have been reported [7-9]. Herein, we present the fourth case of MM complicated by Evans syndrome, which is the first case complicated by both gastrointestinal (GI) bleeding and Evans syndrome.

## Case presentation

A 56-year-old African American male chronic smoker with no significant past medical history presented to the hospital with per rectal bleed of unknown origin (time at presentation [T] = 0 months). His socioeconomic status was poor; however, he had strong family support. The physical examination was unremarkable. Workup including colonoscopy revealed diverticulosis in colon and pancytopenia of unknown cause for which he was transfused multiple times. The patient was discharged subsequently; however, he failed to follow up as an outpatient. Three years later (T = 38 months), he presented again with similar complaints and was admitted for acute GI bleed. Workup on admission revealed worsening of pancytopenia with a WBC count of 3.2 k/ul, hemoglobin of 7 g/dl, and platelet count of 83 k/ul. Computed tomography (CT) scan of the abdomen showed acute diverticulitis for which broad-spectrum antibiotics were initiated, and the patient was subsequently discharged with outpatient esophagogastroduodenoscopy (EGD) and colonoscopy which he never followed up. No apparent cause of pancytopenia could be identified. Three months later (T = 41 months), the patient again presented with abdominal pain and was found to have acute colonic diverticulitis with perforation. General surgery was called, and the patient had sigmoid colectomy with end colostomy (Hartmann's procedure). Biopsies were negative for myeloid protein. 

**Figure 1:**
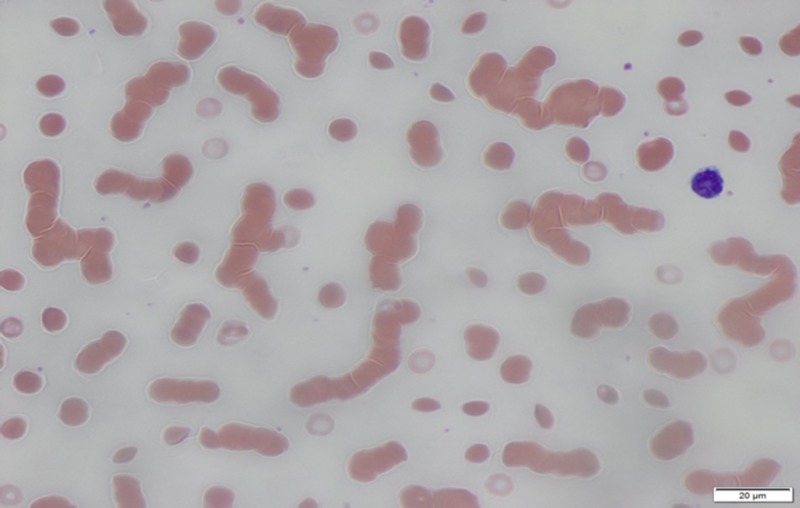
Peripheral smear showing the rouleaux formation Images were taken at 40X resolution.

Post-operatively, the patient continued to present with a similar problem of bleeding from stomas (T= 42 months); multiple re-laparotomies were done with colonic resections due to diverticulitis and multiple small bowel resections due to adhesion formation and strictures. Subsequently, the patient underwent endoscopy again, which revealed antral thickening with biopsy findings as moderate chronic inflammation with goblet cell intestinal metaplasia. Peripheral smear showed rouleaux formation (Figure 1).

**Figure 2:**
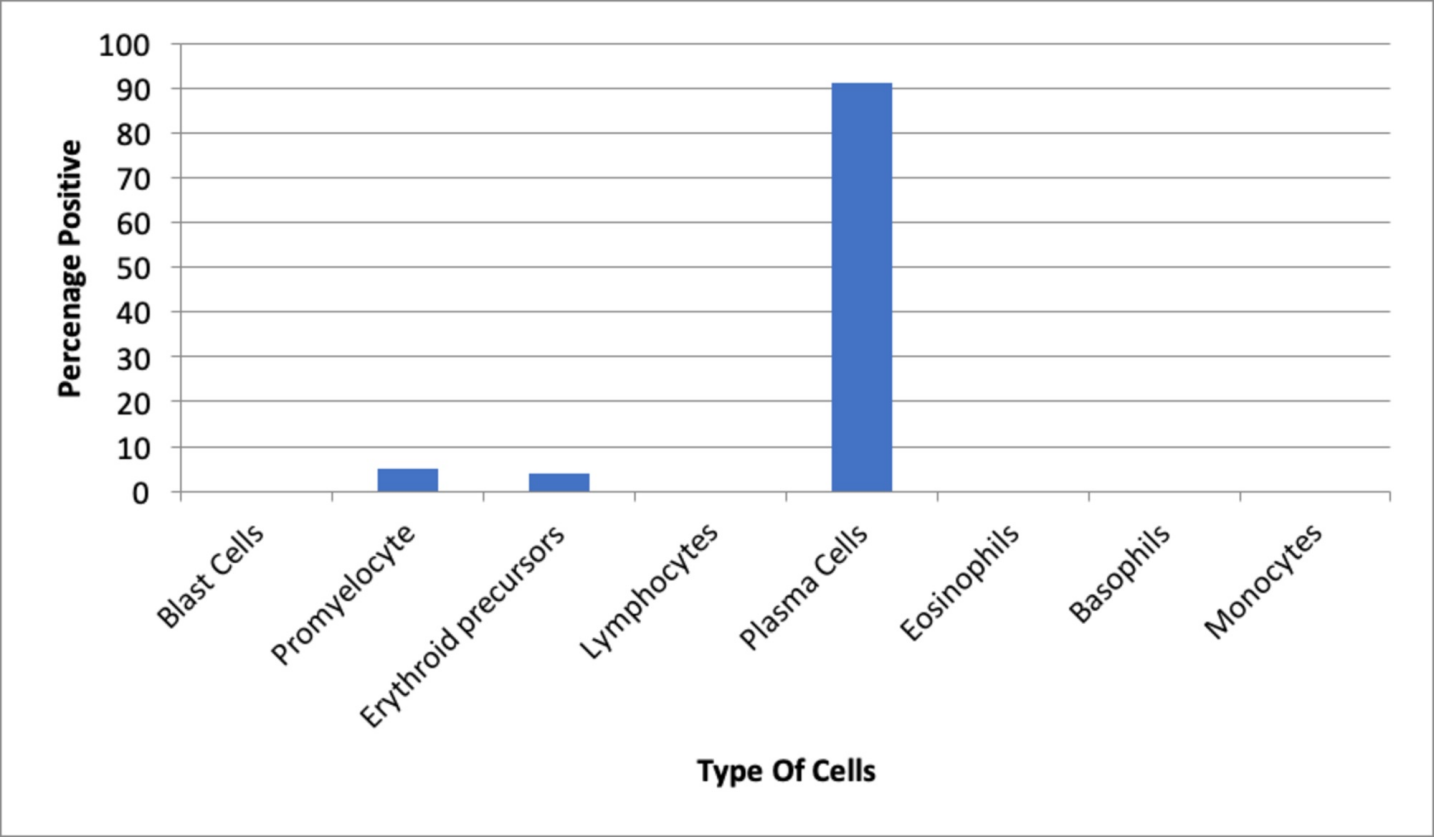
Flow cytometry of bone marrow aspirate showing 91% plasma cells

**Figure 3:**
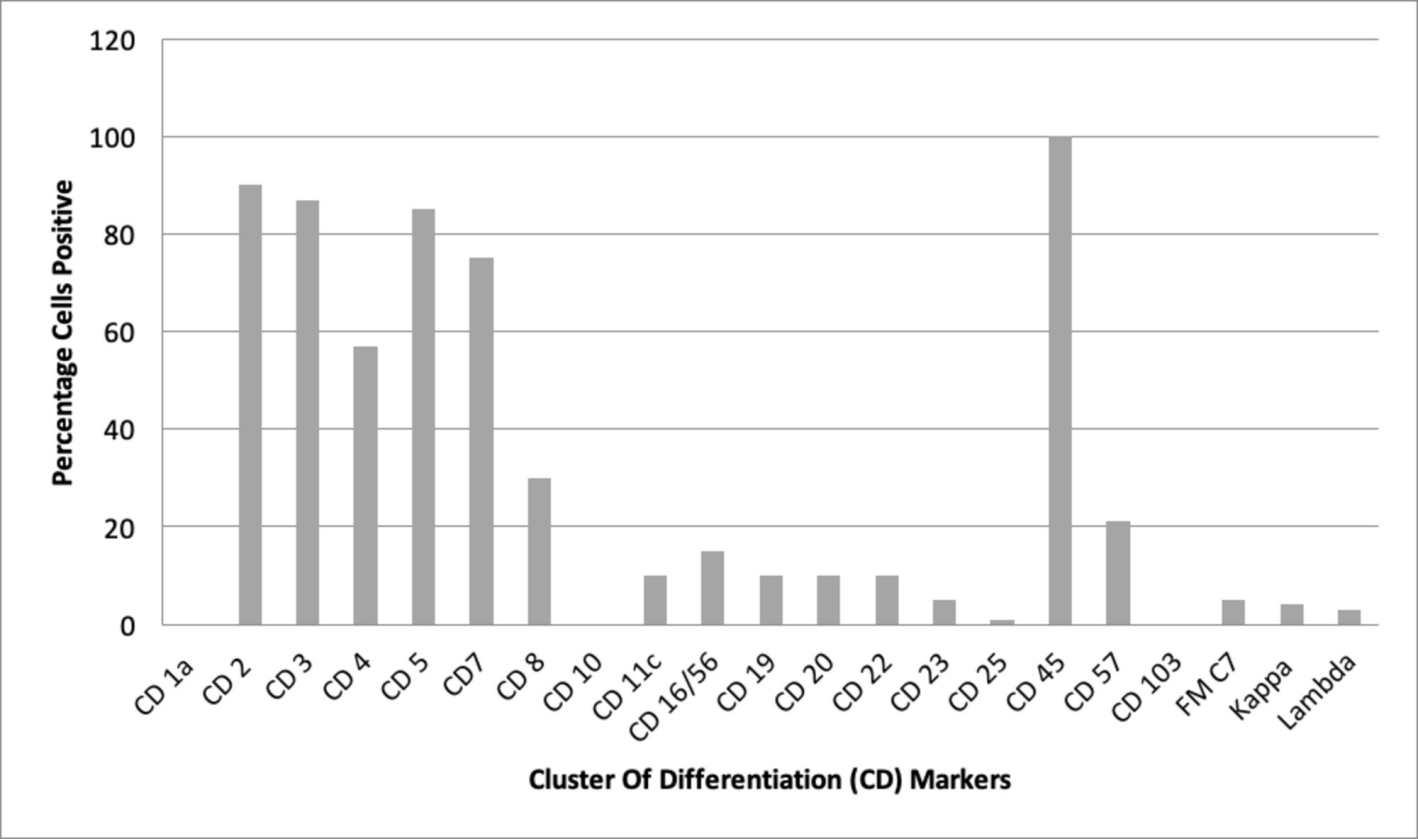
Flow cytometric immunophenotyping analysis performed on bone marrow aspirate following RBC lysis procedure Cells labeled by direct, five color immunostaining procedure and analyzed on an FC500 flow cytometer. *FM C7 is an epitope of CD-20. RBC, red blood corpuscles

Flow cytometry was done for recurrent pancytopenia, which demonstrated increased IgG and abnormal serum kappa/lambda ratio (Figures 2-3).

**Figure 4:**
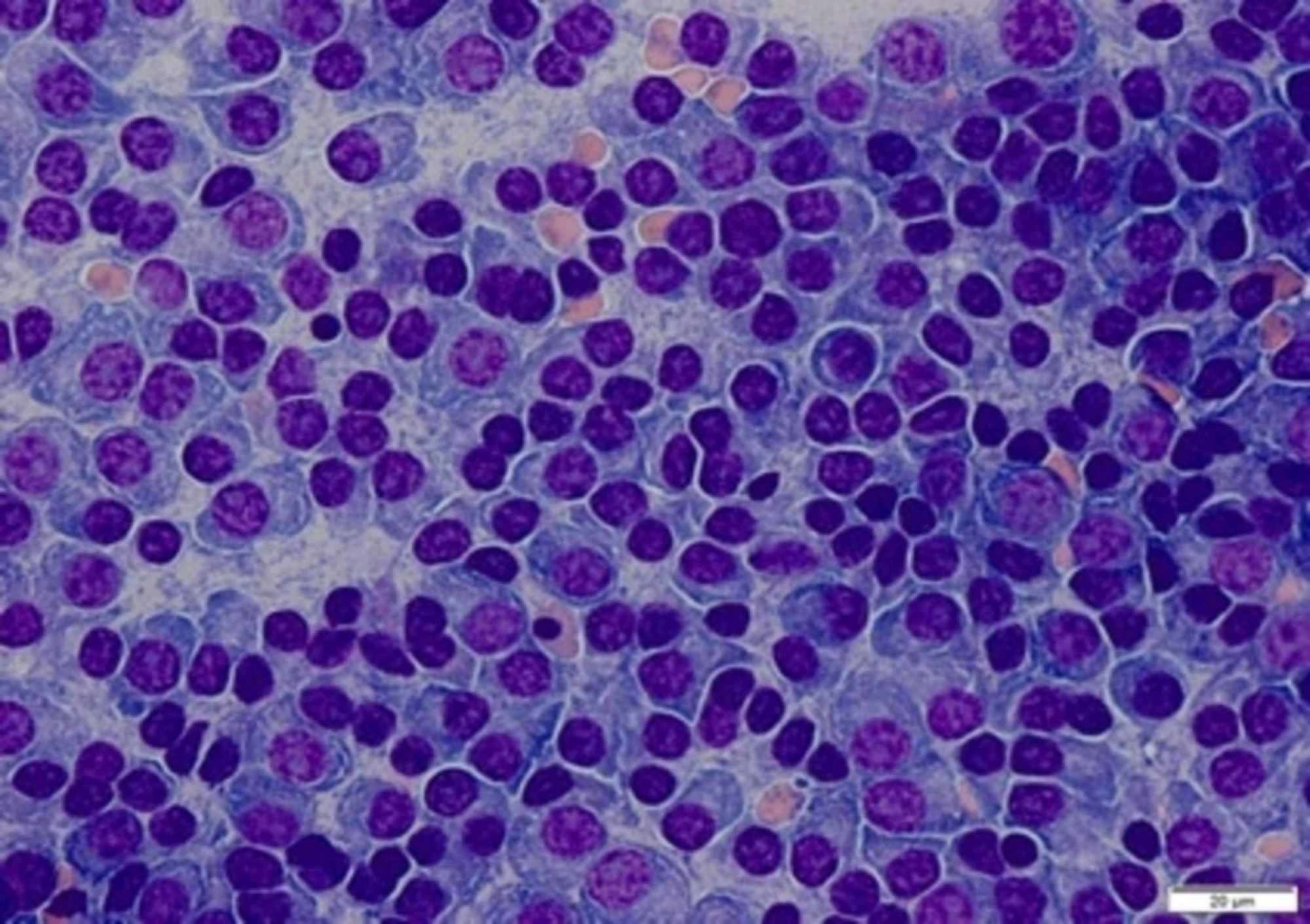
Bone marrow biopsy at 40X resolution showing 80% to 90% plasma cells

The direct antiglobulin test (DAT) yielded positive results. Serum lactate dehydrogenase (LDH) levels were high, and the reticulocyte count was elevated at 2.3% (absolute 0.058), suggesting hemolytic anemia. Serum protein electrophoresis showed paraproteinemia with monoclonal Kappa light chains, suggestive of MM with M protein level of 10.3 g/dl. Bone marrow biopsy revealed 80% to 90% kappa clonal plasma cells confirming MM (Figure 4).

Cytogenetic testing showed 13q deletion and was positive for 1q duplication. Fluorescence in situ hybridization (FISH) was negative for 17p deletion. Small bowel biopsies showed eosinophilic material with mesenteric lymph nodes, suggestive of sinus histiocytosis but negative for amyloid protein. Hematology and oncology were consulted and chemotherapy and plasmapheresis initiated as bortezomib subcutaneous (S/C), continued for 11 cycles, cyclophosphamide intravenous (I/V) for two cycles, changed to oral (P/O) lenalidomide 25 mg daily for a total of 11 cycles. The patient was scheduled to follow-up with rheumatology after being discharged.

Unfortunately, 21 days after transferring to an extended care facility, he presented again (T= 44 months) with chief complaints of generalized body weakness and was admitted with hyperkalemia secondary to acute kidney injury and intravascular hemolysis. Blood workup revealed an elevated total and indirect hyper-bilirubin of 8.10 and 6.8 mg/dl, respectively. Serum LDH was high at 396 U/L, with low haptoglobin of <10 U/L and severe thrombocytopenia 72 k/ul. DAT was positive as of the previous admission. Hematology and oncology recommended high dose I/V methyl prednisone 80 mg TID with I/V hydration due to concern for intravascular hemolysis and autoimmune thrombocytopenia; chemotherapy was put on hold. Over the next few days, he responded to high-dose steroids; repeat blood work showed DAT changing to negative with a normal LDH of 120 U/L and haptoglobin 70 U/L. Thrombocytopenia also reversed to 144 K/ul on discharge despite receiving no platelet transfusions. His hemoglobin and white cell count remained in the low normal range throughout the course of stay but he did require two units of packed red blood cells (RBCs). Oral prednisone was given on discharge with a plan to continue chemotherapy as an outpatient. 

As scheduled, the patient initially had two cycles of CBOrD regimen (T= 46 months) which were changed to bortezomib, lenalidomide, and dexamethasone for a total of 11 cycles. The patient achieved a very good partial response with a 90% reduction in M protein after cycle 3 (T = 48 months). Bone marrow biopsy and aspirate performed after cycle 9 (T = 55 months) did not show any dysmorphia with less than 5% monoclonal plasma cells. Platelet count at cycle 10 (T = 56 months) was stable at 100-130 K /ul; however, he remained chronically anemic likely secondary to bone marrow suppression and silent bleeding from end colostomy. White blood cell count was variable between 2000 and 3000. The plan was to initiate maintenance therapy with lenalidomide PO at T = 57 months. The patient only missed one dose of bortezomib in cycle 7 due to non-compliance. 

Unfortunately, his disease recurred at T = 58 months one month after initiating maintenance therapy. Blood work as outpatient revealed low platelet count at 36 k/ul with DAT turning positive again. Repeat immunofixation studies showed elevated M protein levels consistent with relapse of MM. He was restarted on S/C bortezomib, I/V high dose dexamethasone with a concern for recurring autoimmune hemolysis and thrombocytopenia, and I/V cyclophosphamide for de-bulking to two cycles. 

Two months into the treatment (T= 60 months), he continued to present to the emergency department with complaints of bleeding from his stoma and would leave against medical advice on multiple occasions after receiving blood transfusions. He also continued to follow up as an outpatient with hematology and oncology and continued chemotherapy. His DAT became negative two months after de-bulking chemotherapy; however, pancytopenia persisted and was thought to be multifactorial with evidence of autoimmune hemolysis as evident by extremely low haptoglobin <10 U/L and elevated LDH with a positive DAT. Unfortunately, he had a fall four months into the treatment and was admitted to the intensive care unit and intubated. The computed tomography scan of the brain showed a new-onset sub-arachnoid hemorrhage. The family was consulted about code status this time, and comfort care measures were put in after extensive discussions with poor prognosis in mind. The patient expired after he was extubated. A detailed description of the timeline of events has been tabulated in Table 1.

**Table 1:** Timeline of events DAT, direct anti-globulin test

Time relevant to Initial Presentation in Months (M):	Admitting diagnosis	Intra-hospital Summary
Admission 1: 0 Months	Bleeding due to diverticulosis	Blood transfusions as needed
Admission 2: 38 Months	Acute diverticulitis	Antibiotics with outpatient endoscopy and colonoscopy, Patient failed to follow up as an outpatient.
Admission 3: 41 Months	Colonic perforation secondary to acute diverticulitis	Hartman’s procedure
Admission 4: 42 Months	Post-operative stomal bleed with anemia	Blood transfusions as needed
Admission 5: 42 Months	Recurrent Stomal Bleeds	Multiple laparotomies on the same admission: Pancytopenia worked up; peripheral smear showing rouleaux formation. Flow cytometry for recurrent pancytopenia demonstrating increased IgG and abnormal serum Kappa/Lambda ratio. DAT was positive. Serum lactate dehydrogenase (LDH) and reticulocyte counts were elevated, suggesting hemolytic anemia. Serum protein electrophoresis showing para-proteinemia with monoclonal Kappa light chains, suggestive of multiple myeloma with M protein level of 10.3 g/dl. Multiple myeloma diagnosed with bone marrow biopsy revealing 80% to 90% Kappa clonal plasma cells. Hematology and oncology on board; chemotherapy started outpatient with bortezomib, cyclophosphamide, and dexamethasone (CyBorD). Laparotomy 1: Colectomy with small bowel resections and adhesiolysis. Relook laparotomy 2: Small bowel anastomosis, and end ileostomy. Relook 3: Removal of previous laparotomy pads. Abthera change. Relook 4: Washout and replace abthera. Relook 5: Placement of abdominal facial vicryl mesh, placement of wound vac, placement of abdominal drains.
Admission 6: 43 Months	Altered mental status secondary to dehydration with hypernatremia	Intravenous hydration as needed, continued chemotherapy
Admission 7. 44 Months	Hyperkalemia secondary to acute kidney injury with intravascular hemolysis, likely autoimmune	Intravenous hydration with high dose corticosteroids. Chemotherapy on hold during admission. Platelet 72K on arrival, improve to 144K on discharge, no platelet transfusion given. Peripheral smear shows pancytopenia again with marked rouleau. Direct anti-globulin becomes negative on discharge. Chemotherapy restarted as outpatient.
Admission 8: 45 Months	Bleeding into stoma bag	Blood transfusions as needed
Admission 9. 45 Months	Dizziness due to orthostatic Hypotension due to dehydration	Intravenous hydration. Outpatient events: Two cycles of CyBorD regimen (46 Months) then changed to bortezomib, lenalidomide, and dexamethasone for a total of 11 cycles. Very good partial response achieved after cycle 3.
Admission 10. 46 Months	Pathological compression fracture of T12 and L1 vertebrae	Refused Kyphoplasty. Discharged with physical therapy. Outpatient events: Bone marrow biopsy and aspirate after cycle 9 (55 Months) shows no dysmorphia with less than 5% monoclonal plasma cells. Platelet count at cycle 10 [56 Months] stable at 100-130 K /ul. Plan to start maintenance at cycle 10. Multiple myeloma recurs at 58 months one month after starting maintenance. Debulking therapy initiated.
Admission 11. 58 Months	Atypical chest pain likely musculoskeletal origin	Discharged after a short stay
Admission 12. 59 Months	Epistaxis due to pancytopenia	Three units of packed red blood cell transfusion
Admissions 13,14,15 and 16. 59 and 60 Months	Recurrent stomal bleeding due to pancytopenia	Short stays. Blood transfusions as needed. Refused endoscopy on every admission. Outpatient events: DAT becomes negative two months after de-bulking chemotherapy; however, pancytopenia persists.
Admission 17. 62 Months	Status post fall with subarachnoid hemorrhage	Admitted to the critical care unit. Code status changed due to poor prognosis and expired.

## Discussion

MM is a clonal malignancy of plasma cells producing monoclonal antibodies [6]. In the United States, it presents at a mean age of 71 years in whites and 67 in blacks [6]. Clinically, it is characterized by anemia, hypercalcemia, skeletal lesions and renal failure [8-10]. The incidence of autoimmune diseases, specifically autoimmune hemolytic anemia, in MM remains unclear. One prospective study showed that seven (10.6%) out of 66 patients studied had AIHA [6]. Another literature review revealed almost 4% to 10% of cases having autoimmune hemolytic anemia in MM [11]. The combination of AIHA occurring with immune thrombocytopenia purpura (ITP) is almost rare, and only three cases have been reported up to date [7-9]. Our case is the fourth cause of MM with Evans syndrome and the first associated with recurrent GI bleeds of unknown origin requiring multiple laparotomies. A literature review suggested that pancytopenia, primarily thrombocytopenia, improves with the treatment of primary MM when treated with steroids [6,8-9,11]. 

Our case presented an interesting finding that anemia did not improve with the treatment for primary MM but thrombocytopenia did. Also, the DAT results that showed negative results initially after ten cycles of chemotherapy yielded positive results as MM relapsed. At the time of his death, a negative DAT result was obtained while continuing debulking chemotherapy for the relapsing disease. However, while a negative antiglobulin test correlated with improvement in blood counts, specifically thrombocytopenia in chemotherapy for primary MM, his pancytopenia failed to improve even when DAT was negative in relapsing MM. This is in contrast to a previous prospective study wherein DAT for relapsing MM once positive remained positive during the course of chemotherapy [6]. However, treatment for relapsing myeloma had no effect on the pancytopenia, similar to our study [6].

The reason for the improvement of only one blood cellular lineage might be that the anemia is primarily due to the lack of erythropoietin and hemolysis in MM with marrow infiltration of plasma cells as subordinate in its pathogenesis [1-2]. The thrombocytopenia is usually primarily autoimmune and therefore responds to steroids better than anemia. 

Hence, it might be inferred that DAT provides an indication during the screening for relapse. However, this finding may have limited implications as MM with Evans syndrome or AIHA is an uncommon entity and none of the previous studies except one show any such finding [6]. Furthermore, our case of MM was likely the most aggressive one as compared to the three earlier studies and the first with Evans syndrome to relapse. However, this is an interesting finding never been noticed before in earlier studies. 

Our study is unique in the sense that our patient presented with multiple episodes of bleeding of unknown origin which was never specified. GI bleeding is a presenting symptom in more than 50% of patients with amyloidosis with GI and nonspecific endoscopy findings such as ulcers, erosions, strictures, and hematomas, rarely polyps; however, they are amyloid congo red positive on biopsy [12-13]. In our case, biopsy findings were non-specific ranging from chronic inflammation to intestinal metaplasia; however, they were never positive for amyloid congo red stain or malignancy on repeat laparotomies. Thus, bleeding might have been due to clotting factor deficiencies or thrombocytopenia in MM. MM is also known to be associated with leukocytoclastic vasculitis causing bleeding, although a vessel biopsy was never taken in our case [14]. Whatever the cause may be, abnormal GI bleeding with pancytopenia requiring multiple blood transfusions in otherwise healthy elder individuals may warrant further investigation for MM and autoimmune workup. 

It remains unclear whether our patient had Evans syndrome prior to MM and the chronic inflammation usually related to autoimmune diseases led to clonal blood disorder or his MM led to the production of autoantibodies leading to Evans syndrome. This association needs further workup in the future. However, extensive data will be required before making any hypothesis and considering the extreme rarity of this association, it may take some time before a definitive answer is found.

## Conclusions

In elderly patients with an unknown cause of GI bleeding with pancytopenia, blood dyscrasias especially, MM should be considered and flow cytometry should be performed. In case, the DAT results are positive, steroids can help in improving thrombocytopenia but not other cellular lines.

## References

[1] Beguin Y, Yerna M, Loo M, Weber M, Fillet G (1992). Erythropoiesis in multiple myeloma: defective red cell production due to inappropriate erythropoietin production. Br J Haematol.

[2] Ludwig H, Pohl G, Osterborg A (2004). Anemia in multiple myeloma. Clin Adv Hematol Oncol.

[3] Aguado JM, Castrillo JM, Sanz J, Serrano J (1990). Severe hemolytic anaemia due to cold anti-'i'antibodies associated with cytomegalovirus infection. Postgrad Med J.

[4] Vaiopoulos G, Kyriakou D, Papadaki H, Fessas P, Eliopoulos GD (1994). Multiple myeloma associated with autoimmune hemolytic anemia. Haematologica.

[5] Wada H, Yata K, Mikami M (2004). Multiple myeloma complicated by autoimmune hemolytic anemia. Intern Med.

[6] Kashyap R, Singh A, Kumar P (2016). Prevalence of autoimmune hemolytic anemia in multiple myeloma: a prospective study. Asia Pac J Clin Oncol.

[7] Bechir A, Nesrine BS, Emna B (2016). Multiple myeloma associated with an Evan’s syndrome. The Pan African Medical Journal.

[8] Yi Y, Zhang GS, Gong FJ, Yang JJ (2009). Multiple myeloma complicated by Evans syndrome. Intern Med J.

[9] Al-Ammari Al-Ammari, M. M., & Adam, S. (2016 (2016). Concomitant multiple myeloma, gastric adenocarcinoma and Evan's syndrome in a patient presenting with anemia. BMJ Case Reports.

[10] Landgren O, Zhang Y, Zahm SH, Inskip P, Zheng T, Baris D (2006). Risk of multiple myeloma following medication use and medical conditions: a case-control study in Connecticut women. Cancer Epidemiol Biomark Prev.

[11] Shimanovsky A, Alvarez Argote J, Murali S, Constantin A Dasanu (2016). Autoimmune manifestations in patients with multiple myeloma and monoclonal gammopathy of undetermined significance. BBA Clin.

[12] James DG, Zuckerman GR, Sayuk GS, Wang HL, Prakash C (2007). Clinical recognition of Al type amyloidosis of the luminal gastrointestinal tract. J Gastroenterol Hepatol.

[13] Iida T, Yamano H, Nakase H J (2018). Systemic amyloidosis with gastrointestinal involvement: diagnosis from endoscopic and histological views. J Gastroenterol Hepatol.

[14] Bayer-Garner IB, Smoller BR (2003). Leukocytoclastic (small vessel) vasculitis inmultiple myeloma. Clin Exp Dermatol.

